# Supplementary material for: An Exploration of Pediatricians’ Professional Identities: A Q-Methodology Study
Source: Healthcare (Basel). 2024 Jan 8;12(2):144. doi: 10.3390/healthcare12020144 (PMC10815713; doi:10.3390/healthcare12020144)
Supplement: Supplementary file 1 [file healthcare-12-00144-s001.zip › Table S1 54 final statements.pdf]

Table S1. List of the final Q-set statements (N=54).

1. Keen observation as a professional
2. Ability to discuss problems with parents
3. Ability to explain illness
4. Comprehension skills of children of all ages
5. Ability to judge patients' age and weight
6. Pediatric patient-centered care
7. Ability to judge when a sick child can return to school
8. Learn from others in an open-minded way
9. Emotional management ability
10. Things that kids care about
11. Understand the things kids are interested in
12. Understand the psychological changes of children
13. Understand kids school life
14. Understand the situation of children's families
15. Know the problems that trouble children
16. The ability to soothe children
17. Ability to work together
18. Ability to perform various procedure skills
19. Notice the concerns of family members
20. At the right time to give family a peace of mind
21. Speak in a language that children understand
22. Discuss with family as a work partner
23. Safety climate for patient care
24. Logic of clear thinking
25. Ability to notify patients in advance
26. Pre-announce the possible reaction of examination
27. Being at ease with the assigned teaching and work content
28. Express understanding of the difficulties of family members
29. Use toys or stickers to attract attention for crying patients
30. Perception about the value of colleagues in reducing stress
31. Communicate with colleagues for patients
32. Communication skills to persuade family members and make them approve
33. Can know what emotional response or wording is
34. Resilience
35. Ask family members to accompany the patient when crying
36. Selectively ignore the crying of children
37. Ability to diagnose disease

38. Be patient with family members and sick children
39. Ability to educate patients
40. Ability to explain disease treatment and prognosis
41. Extensive with general knowledge
42. Affinity
43. Teachers and colleagues being at ease with themselves
44. Participate in additional educational and training courses
45. Create trust in parents whose children are hospitalized
46. Physician's research ability
47. Maintain their youthful appearance
48. Communication skills with the family
49. Learned knowledge and spiritual satisfaction from sisters
50. Understanding the emotions of family members will affect doctors' quality of life
51. Centered on lifestyle and well-being
52. Exchange of information for peer benefits
53. Handle different complexities of patients, not only for the very sick ones
54. Dinner with friends and family
