# Supplementary material for: An Exploration of Pediatricians’ Professional Identities: A Q-Methodology Study
Source: Healthcare (Basel). 2024 Jan 8;12(2):144. doi: 10.3390/healthcare12020144 (PMC10815713; doi:10.3390/healthcare12020144)
Supplement: Supplementary file 1 [file healthcare-12-00144-s001.zip › Table S2 Questionnaires.pdf]

Table S2 Questionnaires were administered to each participant during the interviews.

1. Can you explain why these statements are considered most important?
2. Can you explain why these statements are considered least important?
3. For those ranked as most important, can you provide a narrative explanation of their ranking?
4. For those ranked as least important, can you provide a narrative explanation of their ranking?
