# Supplementary material for: An Exploration of Pediatricians’ Professional Identities: A Q-Methodology Study
Source: Healthcare (Basel). 2024 Jan 8;12(2):144. doi: 10.3390/healthcare12020144 (PMC10815713; doi:10.3390/healthcare12020144)
Supplement: Supplementary file 1 [file healthcare-12-00144-s001.zip › Table S4. Factor array for the four factors.pdf]

Table S4. Factor array for the four factors :

Statements	Factor 1	Factor 2	Factor 3	Factor 4
1. Keen observation as a professional	11	10	10	9*
2. Ability to discuss problems with parents	9	10	9	9
3. Ability to explain illness	8	10	10	9
4. Comprehension skills of children of all ages	7	8	9	4
5. Ability to judge patients' age and weight	7	8	6	7
6. Pediatric patient-centered care	8	6	3*	8
7. Ability to judge when a sick child can return to school	8	6	9	5
8. Learn from others in an open-minded way	7	9	10	11
9. Emotional management ability	7	6	4	3
10. Things that kids care about	6	6	5	4*
11. Understand the things kids are interested in	5	7	8	8
12. Understand the psychological changes of children	5	7	8	6
13. Understand kids school life	4	3	3	2
14. Understand the situation of children's families	11	11	6	8
15. Know the problems that trouble children	10	8	5	4
16. The ability to soothe children	11	11	5*	11
17. Ability to work together	8	5	2	7
18. Ability to perform various procedure skills	10	9	11	11
19. Notice the concerns of family members	6	2	8	3
20. At the right time to give family a peace of mind	10*	7	6	5
21. Speak in a language that children understand	9	9	6	4
22. Discuss with family as a work partner	9	9	5*	8
23. Safety climate for patient care	5*	7	8	8
24. Logic of clear thinking	7	4	2	3
25. Ability to notify patients in advance	6	7	7	8
26. Pre-announce the possible reaction of examination	3	5	6	5
27. Being at ease with the assigned teaching and work content	3	3	5	7
28. Express understanding of the difficulties of family members	5	8	7	6
29. Use toys or stickers to attract attention for crying patients	6	4	3	7
30. Perception about the value of colleagues in reducing stress	6	4	7	5
31. Communicate with colleagues for patients	3	5*	7	6
32. Communication skills to persuade family members and make them approve	6	4	9	9

33. Can know what emotional response or wording is	3*	7	6	8
34. Resilience	6	5	9*	6
35. Ask family members to accompany the patient when crying	5	4	3	4
36. Selectively ignore the crying of children	6	2	8	2
37. Ability to diagnose disease	4	3	2	1
38. Be patient with family members and sick children	3*	6	5	6
39. Ability to educate patients	4	1	5	6
40. Ability to explain disease treatment and prognosis	2	1	3	4
41. Extensive with general knowledge	1	3*	1	1
42. Affinity	9	5	4	7
43. Teachers and colleagues being at ease with themselves	8	9	10	9
44. Participate in additional educational and training courses	5	4	8	10
45. Create trust in parents whose children are hospitalized	8	5	6	7
46. Physician's research ability	7	6	7	6
47. Maintain their youthful appearance	4	7	4	5
48. Communication skills with the family	1*	5	5	5
49. Learned knowledge and spiritual satisfaction from sisters	2*	8	7	6
50. Understanding the emotions of family members will affect doctors' quality of life	4	2	1	3
51. Centered on lifestyle and well-being	2*	5	4	5
52. Exchange of information for peer benefits	8	8	7	10
53. Handle different complexities of patients, not only for the very sick ones	5	3	4	3
54. Dinner with friends and family	4	6	4	2
